# CKS2 Silencing Affects Proliferation and Apoptosis in Multiple Myeloma through the PTEN/ AKT/mTOR Pathway

**DOI:** 10.7150/jca.106190

**Published:** 2025-03-03

**Authors:** Jing Zi-zi, Yu Wei, Tang Jia-lin, Zhou Xiao-bin, Chen Jian-bin

**Affiliations:** Department of Hematology, The First Affiliated Hospital of Chongqing Medical University, Chongqing, China.

**Keywords:** CKS2, multiple myeloma, PTEN, TXN, apoptosis

## Abstract

Multiple myeloma (MM), a prevalent plasma cell malignancy, represents a life-threatening hematological disorder with significant clinical morbidity. Despite its recognized impact on global health burdens, the precise molecular pathogenesis underlying disease progression remains incompletely elucidated. Transcriptomic profiling via RNA sequencing revealed significant upregulation of cyclin-dependent kinase regulatory subunit 2 (CKS2) in multiple myeloma. Clinical validation was performed through quantitative analysis of CKS2 expression in patient-derived specimens. Two established MM cell models (MM.1S and RPMI-8226) were selected for functional characterization. Cellular proliferation dynamics were quantified using CCK-8 metabolic assays and EdU DNA incorporation analysis, with flow cytometric evaluation employed to assess apoptotic indices. A xenograft mouse model was established to investigate CKS2-mediated tumorigenesis *in vivo*, complemented by western blot analysis of pathway-associated protein expression. Bioinformatic interrogation of the HumanBase database identified putative CKS2 interactomes, subsequently validated through co-immunoprecipitation assays and confocal immunofluorescence microscopy. Structural modeling via AlphaFold2 predicted molecular interaction interfaces, with three-dimensional visualization achieved through PyMOL rendering. In this study, we demonstrated that CKS2 knockdown in MM.1S and RPMI-8226 cell lines significantly inhibited cellular proliferation and induced apoptosis. Conversely, CKS2 overexpression enhanced malignant proliferation while suppressing apoptotic processes, establishing its functional role in myeloma pathogenesis. Mechanistic investigations revealed that CKS2 depletion modulates cell proliferation and apoptosis via PTEN/AKT/mTOR signaling axis. Notably, co-immunoprecipitation assays demonstrated direct protein-protein interaction between CKS2 and thioredoxin (TXN), with subsequent functional validation suggesting TXN appears to function as a key upstream regulatory factor governing CKS2 stability. These findings establish CKS2 as a critical regulator of myeloma cell homeostasis and identify it as a promising therapeutic target warranting further preclinical validation.

## Introduction

Multiple myeloma (MM), a hematologic malignancy originating from clonal plasma cell proliferation in the bone marrow, represents a significant global health burden with an estimated annual incidence of 588,161 cases worldwide^1^. MM is characterized by excessive clonal expansion of malignant plasma cells within the bone marrow microenvironment. This pathological process disrupts normal hematopoiesis, leading to anemia through suppression of erythroid progenitor cells. The neoplastic infiltration induces osteoclast activation, resulting in osteolytic bone lesions with associated pathological fractures and secondary hypercalcemia. These skeletal complications frequently coexist with renal impairment due to: (1) calcium-mediated nephropathy; (2) monoclonal immunoglobulin (M protein) deposition in renal tubules; and (3) light chain proteinuria. Furthermore, compromised humoral immunity from defective antibody production significantly elevates infection risk in MM patients^2^.

The current standard first-line regimen for multiple myeloma integrates a multidrug approach combining three principal therapeutic classes: proteasome inhibitors (e.g., bortezomib), immunomodulatory agents (e.g., lenalidomide), and monoclonal antibodies (e.g., daratumumab), typically administered with corticosteroids like dexamethasone as combination therapy. For eligible patients, autologous stem cell transplantation (ASCT) remains a cornerstone consolidative intervention. Emerging modalities such as chimeric antigen receptor T-cell (CAR-T) therapy demonstrate promising efficacy in refractory cases. Following induction therapy, maintenance protocols employing low-dose regimens (commonly lenalidomide or bortezomib-based) are implemented to sustain remission and improve long-term disease control, with contemporary studies confirming significant progression-free survival benefits^3^, ^4^. While significant therapeutic progress has been achieved in managing MM, the disease remains incurable due to persistent challenges in eradicating drug-resistant clones. Furthermore, the precise pathogenesis of MM - particularly the interplay between genetic instability, epigenetic dysregulation, and bone marrow microenvironment interactions - continues to elude complete scientific understanding.

In this investigation, we performed a comparative transcriptomic analysis of MM versus healthy donor samples leveraging the GEO repository (GSE6477, GSE47552). Subsequent multimodal validation encompassing clinical specimens and functional assays demonstrated that CKS2 modulates MM pathobiology via the PTEN/AKT/mTOR signaling axis. Notably, integrated protein interaction network analysis revealed direct binding affinity between TXN and CKS2, as validated by co-immunoprecipitation assays combined with computational docking simulations. These findings position CKS2 as a potential therapeutic target for multiple myeloma management.

## Method and materials

### Patient datasets and clinical samples

The mRNA expression profiles and corresponding clinical metadata of multiple myeloma (MM) patients were retrieved from the Gene Expression Omnibus (GEO) database. All samples corresponded to newly diagnosed untreated patients and classified according to the International Myeloma Working Group criteria. Two independent cohorts were analyzed: GSE47552 (n=41 MM vs. 5 healthy controls) and GSE6477 (n=73 MM vs. 15 healthy controls) for differential gene expression analysis, while the GSE24080 cohort (n=559) provided comprehensive clinical annotations for survival correlation studies. The clinical samples were collected by bone marrow aspiration at the first affiliated hospital of Chongqing Medical University. Patients were diagnosed with multiple myeloma according to the International Myeloma Working Group (IMWG) and had not received any treatment. The inclusion criteria as follows^5^: clonal bone marrow plasma cells ≥10% or biopsy-proven osseous or extramedullary plasmacytoma and any one or more of the following myeloma-defining events. Myeloma defining events conclude: 1. evidence of end organ damage that can be attributed to the underlying plasma cell proliferative disorder, specifically hypercalcemia, renal insufficiency, anemia and bone lesions. 2. Any one or more of the following biomarkers of malignancy: I. clonal bone marrow plasma cell percentage ≥60%, II. Involved: uninvolved serum free light chain ratio ≥100, III. >1 focal lesion on MRI studies. The related clinical characteristics of MM patients were also collected. 16 patients with iron deficiency anemia were collected as the normal control. Informed consent was obtained from each patient. The study protocol was approved by the Ethics Committee of the First Affiliated Hospital of Chongqing Medical University (approved number: K2023-234).

### Real-time quantitative polymerase chain reaction

Following bone marrow aspiration, mononuclear cells were isolated through Ficoll-Paque density gradient centrifugation. Total RNA was subsequently extracted from the isolated cells using TRIzol™ reagent and reverse-transcribed into complementary DNA (cDNA) with the MCE Reverse Transcription System. Quantitative real-time PCR amplification was performed by using the SYBR Green Fast Real-Time PCR system, with all primers synthesized and sequence-validated by Tsingke Biotechnology Co., Ltd. (Beijing, China). CKS2-forward: 5'-TTCGACGAACACTACGAGTACC-3'; CKS2-reverse: 5'-GGACACCAAGTCTCCTCCAC-3'; GAPDH-forward: 5'-GGAGTCCACTGGCGTCTTCA-3'; GAPDH-reverse: 5'-GTCATGAGTCCTTCCACGATACC-3'.

### Cell culture

Human multiple myeloma cell lines MM.1S and RPMI-8226 were purchased from the cell bank of Shanghai and Beijing, Chinese Academy of Science. All cells were cultured in RPMI 1640 media supplemented with 10% fetal bovine serum (PAN) and 1% Penicillin-Streptomycin Solution (Beyotime). The cells were incubated at 37°C with 5% 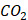.

### Cell transfection

Two siRNA targeting CKS2 were used in the study, including CKS2 siRNA (RNAi#1, 5'-GTCTAGGCTGGGTTCATTA-3' and RNAi#2, 5'-CCTGTGCATGAGCTGTATT-3'). Negative siRNA was used as the control group. The lentiviral vectors were purchased from Tsingke. The coding sequences of overexpressed human CKS2 were cloned into PLO100 (GFP and Puro) vector. The shRNA of lentivirus targeting the human CKS2 mRNA sequence (5'- GUCUAGGCUGGGUUCAUUA-3') were cloned into pLVX (GFP and Puro) vector. The shRNA of lentivirus targeting the human TXN mRNA sequence (5'- GACTGTCAGGATGTTGCTTCAGAGTGTGA-3') was cloned into plko.1 (GFP and Puro) vector. Transfection was carried out according to the manufacturer's instructions.

### CCK-8 assay

Adjust the cell density to 1x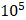/ ml then put 100ul of cell suspension into the 96-well plate and the cells were cultured for 0, 24, 48, and 72 hours, add 10ul of CCK-8 working reagent, continue incubation for 1 hour, and then use an enzyme reader to detect the absorbance at a wavelength of 450nm.

### EdU assay

Cells were incubated with EdU labeling solution for 2-3 h at 37°C. Following incubation, cells were harvested and fixed with methanol-acetic acid fixative (3:1 v/v) for 30 min at room temperature. Cell slides were prepared and subsequently baked at 42°C for 30 min. Slides underwent sequential processing as follows: 1. Three 5-min PBS rinses. 2. Permeabilization with 0.5% Triton X-100 (10 min, RT). 3. Three additional 5-min PBS washes. 4. Click reaction using a freshly prepared EdU detection cocktail (30 min, dark conditions). 5. Nuclear counterstaining with DAPI (10 min, RT protected from light). 6. Final PBS wash cycle (3×5 min), spin dry the slides and seal with an anti-fluorescence quenching sealing solution. A fluorescence microscope was used to observe and take pictures.

### Cell apoptosis detection

The collected MM cells were washed twice with PBS, and a small amount of PBS buffer was left to resuspend the cells, and the mixture was gently blown. Take 100ul of cell suspension, add 5ul Annexin V-APC and 5ul DAPI, mix well, incubate at room temperature in the dark for 15min, and wash once with PBS. Resuspend the cells with 500ul PBS buffer and detect them on the flow cytometry.

### Western blot

MM cells were collected and washed three times with PBS. Protein lysis mixture (RIPA: PMSF: phosphatase inhibitor = 100:1:2) was added to lyse the cells, lysed on ice for 30 minutes, centrifuged at 4°C, 12000g for 20 minutes, harvested the supernatant, added loading buffer and boiled for 10 minutes. Protein samples were separated by SDS-PAGE gel and transferred onto the PVDF membrane, blocked with 5% skim milk powder at room temperature for 1 hour, incubated with primary antibody overnight; washed with TBST (3 times x 10 minutes), incubated with secondary antibody at room temperature for 1 hour, washed with TBST (3 times x 10 minutes), and finally detected immunoreactive protein bands by chemiluminescence.

### Xenograft tumor models

Male nude mice (5 weeks) were purchased from Chongqing Enswell Biotechnology Co., Ltd. and housed in the IVC laboratory of the school's experimental animal center, with good ventilation, sufficient feed, and clean drinking water. The nude mice were randomly divided into two groups (sh-CKS2 group and sh-NC group), and the cells were injected subcutaneously at the back of the nude mice's neck. The amount of cells inoculated per nude mouse was 1x10^7/0.2 ml. The general conditions of the nude mice, such as urination, diet, mental state, and subcutaneous tumor formation were observed and recorded. After 5 weeks, the mice were sacrificed, and the tumors were removed and photographed. The animal experiment protocol complies with the principles of animal protection, animal welfare and ethics, and the relevant provisions of the national laboratory animal welfare ethics. It has been approved by the Laboratory Animal Management and Experiment Committee of Chongqing Medical University (approval number: IACUC-CQMU-2023-0025).

### Immunohistochemistry (IHC)

Immunohistochemistry (IHC) assay was performed as previously described^6^. Bone marrow biopsy samples and nude mouse subcutaneous tumors were fixed with 4% paraformaldehyde (bone marrow biopsy samples were decalcified before fixation), dehydrated, embedded in paraffin, and then prepared into 4 mm thick sections. The sections were baked in a 60 ° C oven for 2 hours, and then further dewaxed with xylene and gradient alcohol, and microwaved for 5 minutes on high heat and 15 minutes on low heat for antigen repair. After cooling to room temperature, goat serum was used for blocking, and diluted primary antibodies were added and incubated overnight at 4°C. The secondary antibody was incubated at room temperature for 1 hour, DBA was used for color development, hematoxylin was used for counterstaining, and then dehydrated with gradient alcohol and xylene, and the sections were sealed with neutral resin. Finally observed under an optical microscope. IHC antibody: CKS2(1:200, #AF0616, Affinity), Ki-67 (1:200, #12202T; CST), PCNA (1:100, #WL03213; Wanlei).

### Co-immunoprecipitation

MM cells were collected and washed three times with PBS, lysed with NP-40 on ice, centrifuged at 12000g, 4°C for 20min, and collected supernatant. Divide the supernatant into 3 parts, one as the Input group, add loading buffer, boil, and store at -20°C; one as the IgG group, and one as the IP group, add 4ug IgG antibody and 4ug anti-TXN antibody respectively, and rotate at 4°C overnight. Add 25ul magnetic beads, mix well, rotate at 4°C for 4h, wash 3 times with NP-40 lysis buffer on the magnetic stand, and wash with distilled water for the last time, discard all liquid, add loading buffer, boil for 10min, and transfer the liquid to a new ep tube on the magnetic stand. Subsequent detection methods are the same as western blot.

### Immunofluorescence

Put the cell slides into a 6-well plate and coat the slide with the cell adhesion agent (Applygen Technologies). Inoculate the cells into the six-well plate and continue to culture. When the appropriate cell density is reached, discard the culture medium in the six-well plate, add PBS buffer to wash twice, discard PBS, add 4% paraformaldehyde to fix the cells at room temperature for 20 minutes, discard paraformaldehyde, add PBS buffer, place the six-well plate on a shaker and shake slowly to wash (3 times x 3 minutes), discard PBS buffer; add 0.5% Triton-X100 to permeabilize at room temperature for 10 minutes, add PBS buffer to wash (3 times x 3 minutes), discard PBS buffer; block with 1% BSA at room temperature for 30 minutes; dilute the immunofluorescence primary antibody (anti-CKS2, Rabbit, 1:200, anti-TXN, Mouse, 1:50), evenly cover the immunofluorescence primary antibody on the cell slide, incubate at 4°C overnight; rewarm at room temperature for 30 minutes, gently wash with PBS buffer (3 times x 3 minutes); dilute the fluorescent secondary antibody, evenly cover the surface of the cell slide, incubate at room temperature in the dark for 1 hour; wash with PBS buffer (3 times x 3 minutes); add DAPI to stain the nucleus, incubate at room temperature in the dark for 10 minutes; wash with PBS buffer (3 times x 3 minutes); finally, absorb excess water from the surface of the cell slides, seal the slide with anti-fluorescence quenching sealant, observe and photograph under a confocal microscope.

### Statistical analysis

Statistical analysis was performed using IBM SPSS Statistics 26 and GraphPad Prism version 8.0 for Windows. Data were expressed as the Means ± SEM. Student's t-test was used to analyze the differences between the two groups. One-way ANOVA was used to analyze the differences between more than two groups. The Chi-square test and T-test were used to examine the relationship between CKS2 and clinicopathological characteristics. Kaplan-Meier survival analysis was used to analyze overall survival (OS) time. *p*<0.05 means statistically significant.

## Results

### CKS2 is highly expressed in MM and is associated with poor prognosis

**Figure 1:**
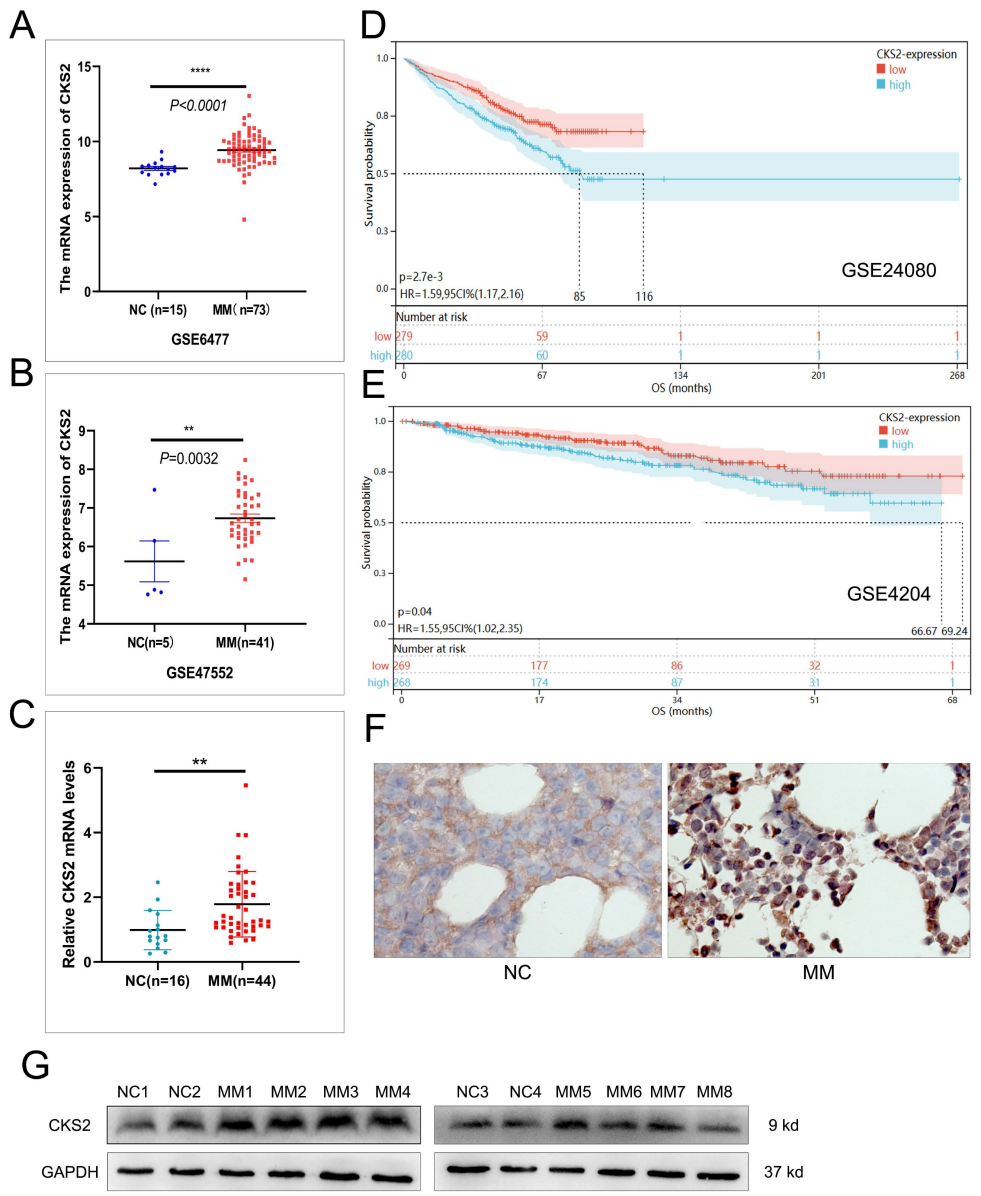
** CKS2 is highly expressed in multiple myeloma.** A and B: The mRNA expression of CKS2 in the GSE6477 and GSE47552 datasets; C: RT-qPCR was used to detect the relative expression of CKS2 mRNA in clinical bone marrow specimens. D and E: Kaplan-Meier (K-M) survival analysis in GSE24080 and GSE4204 datasets. F and G: Immunohistochemistry and Western blot were used to detect the CKS2 expression in specimens. IHC magnification: X200. Data are shown as mean ± SEM. **p<0.01, ****p<0.0001.

We initiated our investigation by analyzing RNA-seq data from the GEO repository (accessions GSE6477 and GSE47552), comparing bone marrow samples of multiple myeloma (MM) patients with healthy controls. Bioinformatics analysis revealed significant upregulation of CKS2 mRNA levels in MM cohorts across both datasets (Figure 1A-B). To validate these findings, we performed RT-qPCR on bone marrow aspirates from 44 newly diagnosed MM patients and 16 iron-deficiency anemia controls, confirming elevated CKS2 transcript levels in MM samples (Figure 1C). Western blot and IHC analysis demonstrated concordant overexpression of CKS2 protein in MM patients versus controls (Figure 1F-G).

Prognostic relevance was assessed through Kaplan-Meier analysis of survival-associated GEO datasets (GSE24080, GSE4204), revealing significantly shorter overall survival (OS) in MM patients with high CKS2 expression (log-rank p<0.05, Figure 1D-E). Further interrogation of the GSE24080 dataset (n=559) identified significant correlations between CKS2 expression and established prognostic markers: elevated ISS stage, β2-microglobulin (B2M), C-reactive protein (CRP), lactate dehydrogenase (LDH), and MRI-defined focal lesion burden, alongside reduced albumin (ALB) and hemoglobin (HGB) levels (Table 1). Multivariate Cox regression analysis established CKS2 as an independent prognostic factor for MM survival (HR=1.403, 95% CI:1.028-1.914, p=0.033, Table 2). Supplementary analysis of our 44-patient cohort reinforced these findings (Supplementary Tables 1-2). Collectively, our multi-modal evidence positions CKS2 as a clinically significant biomarker associated with poor outcomes.

### CKS2 regulates MM cell proliferation

**Figure 2:**
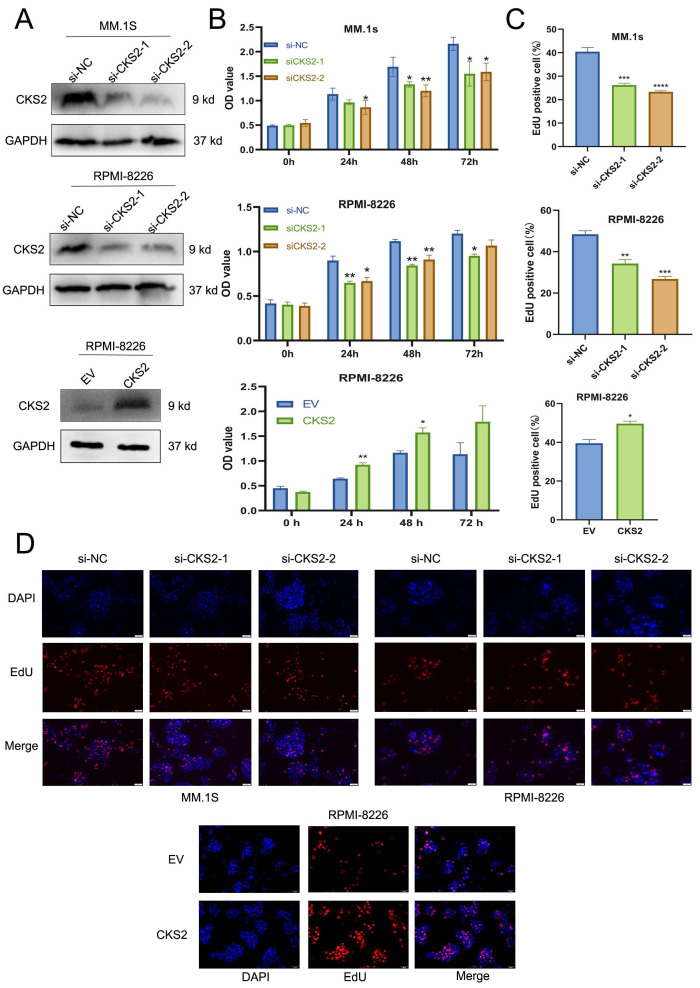
** CKS2 affects MM cell proliferation *in vitro*.** A. siRNA was used to silence CKS2 in MM.1S and RPMI-8226 cells, and CKS2 was overexpressed in RPMI-8226 cells. B. Cell viability was measured after CKS2 knockdown or overexpression via CCK-8 assay. C and D. Edu assay detected the DNA replication in MM.1S and RPMI-8226 cells. Data are shown as mean ± SEM. *p<0.05, **p<0.01, ***p<0.001, ****p<0.0001.

CCK-8 assay was used to assess MM cell viability. The results revealed that compared with the control group, the cell viability of the CKS2 knockdown group was significantly reduced; while the viability of MM cells with CKS2 overexpression increased (Figure 2B). The EdU assay was used to quantify cellular DNA replication. The results showed that compared with the control group, DNA replication of cells in the CKS2 knockdown group was inhibited, whereas DNA replication of MM cells in the CKS2 overexpression group increased (Figure 2C-D). The above results collectively demonstrated that knockdown of CKS2 inhibits MM cell proliferation, while overexpression of CKS2 promotes MM cell proliferation.

### CKS2 regulates MM cell apoptosis

**Figure 3:**
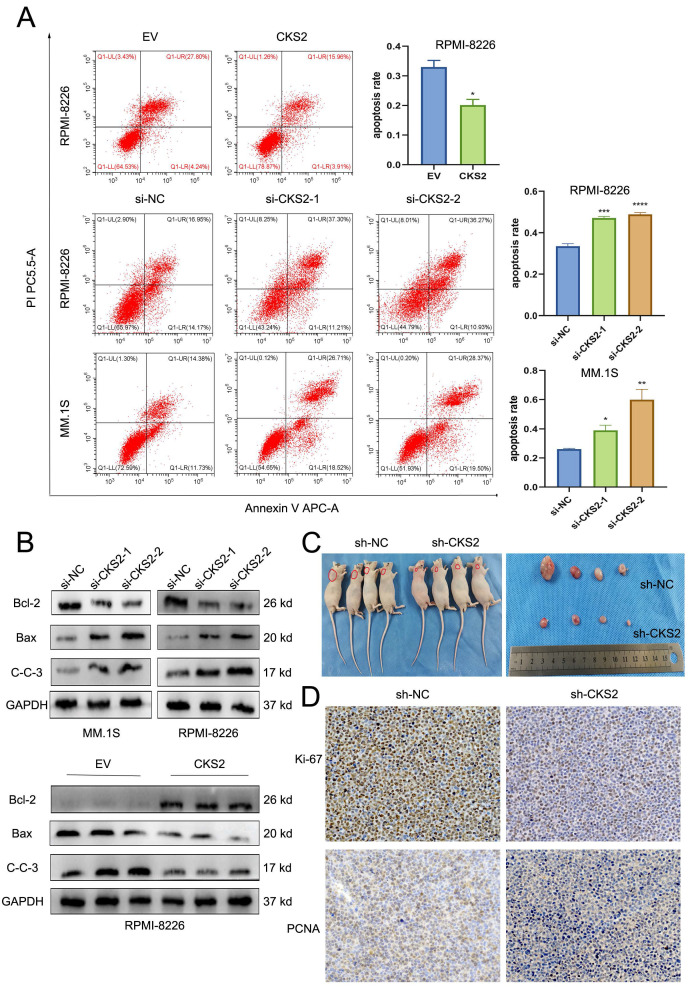
** CKS2 affects MM cell apoptosis *in vitro* and proliferation *in vivo*.** A. Flow cytometry measured the cell apoptosis rate in MM.1S and RPMI-8226 cells. B. Western blot detected the expression of apoptosis-related proteins in MM.1S and RPMI-8226 cells. C. Xenograft tumor was photographed after the mice were sacrificed. D. Immunohistochemistry staining detected the expression of Ki-67 and PCNA in xenograft tumor tissue sections, magnification: X200. Data are shown as mean ± SEM. *p<0.05, ***p<0.001, ****p<0.0001.

To explore the effect of CKS2, flow cytometry was used to assess the apoptosis of MM cells. After knocking down of CKS2, the apoptosis rate of MM cells was significantly higher than that of the control group; while after overexpressing CKS2, the apoptosis rate of MM cells was reduced (Figure 3A). In addition, we also detected the expression of apoptosis-related proteins by Western blot. The results showed that after the knocking down of CKS2, the expression of pro-apoptosis-related proteins Bax and Cleaved-Caspase 3 increased, while the expression of anti-apoptosis-related protein Bcl-2 decreased. After overexpression of CKS2, the change trend of apoptosis-related proteins was just the opposite (Figure 3B). The above results demonstrated that knocking down CKS2 can promote MM cell apoptosis while overexpressing CKS2 can inhibit MM cell apoptosis.

### Knockdown of CKS2 inhibits MM proliferation *in vivo*

To further verify the effect of CKS2 expression on the proliferation of MM cells *in vivo*, we constructed an RPMI-8226 cell line with stable knockdown of CKS2 and constructed an MM xenograft tumor nude mouse model by subcutaneous tumor formation. 5 weeks after cell inoculation, the nude mice were sacrificed, and the tumors were peeled off and photographed (Figure 3C); immunohistochemical staining of xenograft tumor tissues revealed that the expression of Ki-67 and PCNA proteins in the CKS2-knockdown cohort was reduced compared with the control group (Figure 3D). The above results revealed that the knockdown of CKS2 can inhibit the proliferation of MM cells *in vivo*.

### CKS2 regulates tumor-related AKT-mTOR pathway

**Figure 4:**
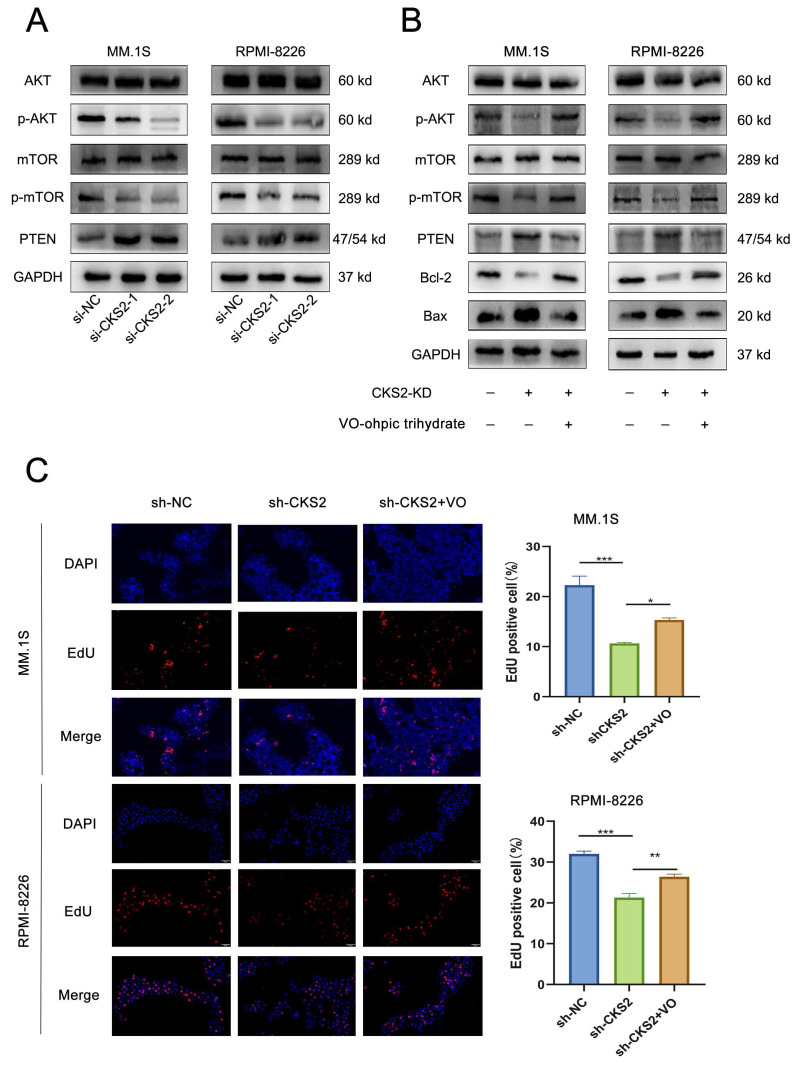
** CKS2 affects the AKT/mTOR signaling pathway through PTEN.** A. Western blot was used to detect the expression of related proteins after CKS2 knockdown. B. PTEN inhibitor reversed the expression of p-AKT/p-mTOR and apoptosis-related proteins induced by CKS2 knockdown. C. EdU assay measured the DNA replication after the PTEN inhibitor treatment in MM.1S and RPMI-8226 cells. Data are shown as mean ± SEM. *p<0.05, **p<0.01, ***p<0.001.

Based on these findings, we conclude that CKS2 modulates biological functions in multiple myeloma (MM) cells. To investigate the underlying mechanism, we performed comprehensive literature review and experimental validation. Notably, CKS2 knockdown in MM cells resulted in upregulated PTEN expression. As a canonical tumor suppressor, PTEN is critically involved in cellular processes including growth regulation, signal transduction, and apoptotic control. Western blot analyses revealed concomitant downregulation of phosphorylated AKT (p-AKT) and mTOR (p-mTOR) following CKS2 depletion (Figure 4A). To establish the PTEN-dependent nature of this regulation, we employed the specific PTEN inhibitor VO-Ohpic trihydrate (HY-13074, MCE) in CKS2-knockdown cells. The inhibitor effectively reversed both the phosphorylation changes of AKT/mTOR and the alterations in apoptosis-related protein expression induced by CKS2 knockdown (Figure 4B). Furthermore, PTEN inhibition rescued the anti-proliferative effects associated with CKS2 depletion (Figure 4C). Collectively, these data demonstrate that CKS2 regulates MM cell functionality through the PTEN/AKT/mTOR signaling axis.

### TXN interacts with CKS2 to regulate the AKT-mTOR pathway

**Figure 5:**
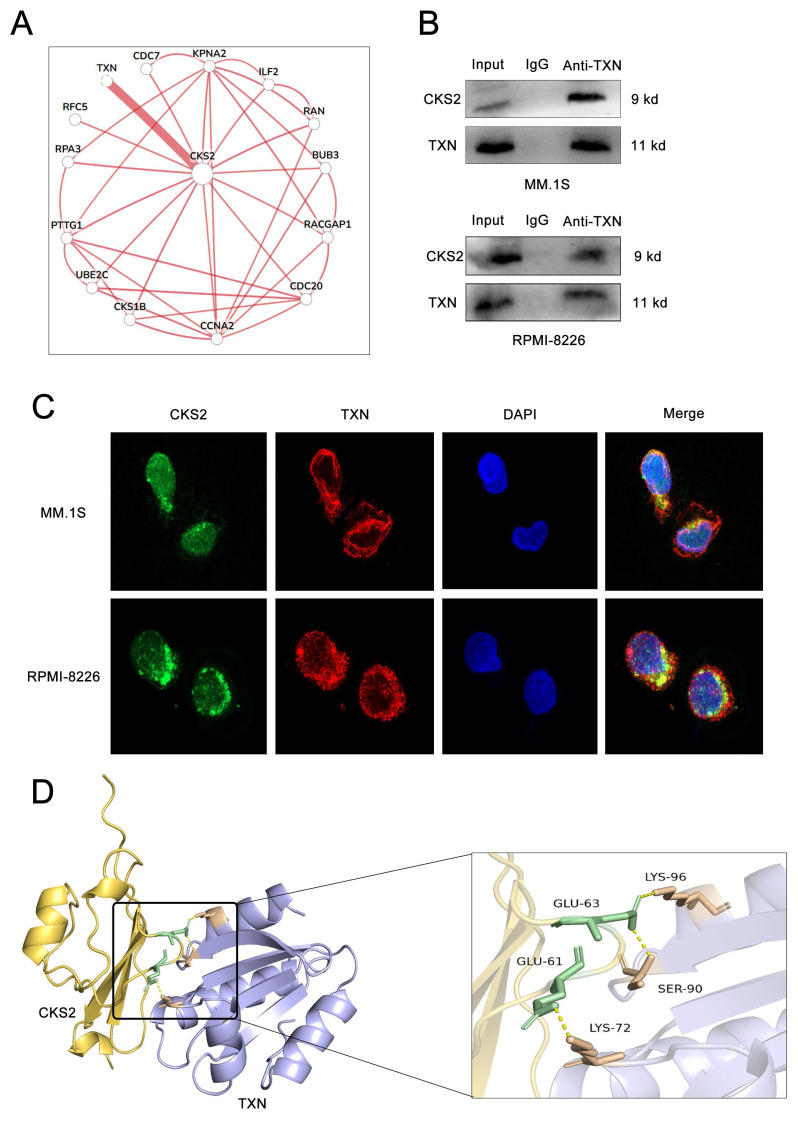
** CKS2 interacts with TXN.** A. 14 Factors predicted by bioinformatics that may interact with CKS2. B. Co-immunoprecipitation (Co-IP) was used to measure the interaction between CKS2 and TXN. C. Immunofluorescence detected the co-localization of CKS2 and TXN in MM.1S and RPMI-8226 cells, magnification: X630. D. PyMol was used to visualize the interaction between CKS2 and TXN.

Using the HumanBase protein-protein interaction prediction platform, we identified 14 potential CKS2-interacting factors (Figure 5A). Subsequent prioritization revealed thioredoxin (TXN) as a candidate showing significant differential expression between multiple myeloma (MM) patients and controls, with elevated TXN levels observed in MM specimens (Supplemental Table 3).

The CKS2-TXN interaction was confirmed through co-immunoprecipitation assays (Figure 5B) and further validated via immunofluorescence co-localization, demonstrating cytoplasmic colocalization in MM cells (Figure 5C). Structural modeling using AlphaFold Server (v2.3.2) with PyMOL visualization predicted specific molecular interactions: CKS2-Glu61 with TXN-Lys72, and CKS2-Glu63 with TXN-Ser90/Lys96 (Figure 5D).

**Figure 6:**
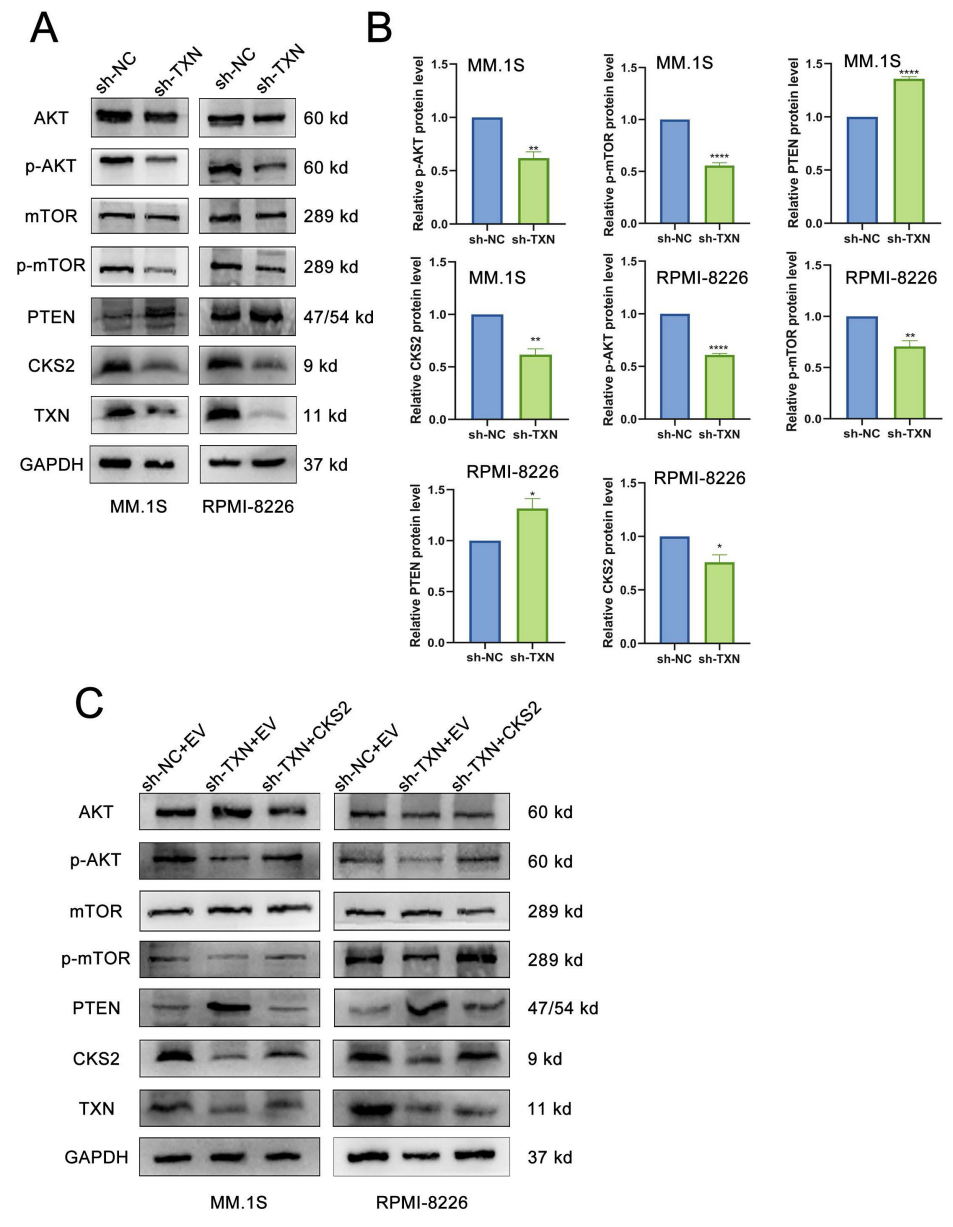
** TXN interacts with CKS2 to regulate the AKT-mTOR pathway.** A and B. Western blot was used to detect the expression of CKS2, PTEN, AKT, and mTOR after TXN knocking down in MM.1S and RPMI-8226 cells. C. Overexpression CKS2 reversed the expression of PTEN, p-AKT, and p-mTOR induced by TXN knocking down in MM.1S and RPMI-8226 cells. Data are shown as mean ± SEM. *p<0.05, **p<0.01, ****p<0.0001.

Following TXN knockdown in MM cells, western blot revealed a downregulation of CKS2 expression, concomitant with increased PTEN levels and reduced phosphorylation of AKT (p-AKT) and mTOR (p-mTOR) (Figures 6A-B). Subsequent rescue experiments demonstrated that CKS2 overexpression in TXN-deficient MM cells effectively rescued the TXN knockdown-induced alterations in PTEN expression and AKT/mTOR phosphorylation expression (Figure 6C).

## Discussion

Multiple myeloma (MM), a hematological malignancy of unclear etiology, primarily relies on chemotherapeutic regimens as first-line treatment, with hematopoietic stem cell transplantation recommended for eligible patients. Elucidating the molecular mechanisms underlying MM pathogenesis remains clinically imperative. Our investigation revealed elevated CKS2 expression in bone marrow samples from MM patients compared to healthy controls, with high CKS2 expression correlating with reduced overall survival. Functional studies demonstrated that CKS2 knockdown significantly inhibited MM cell proliferation and enhanced apoptosis. Mechanistically, CKS2 depletion upregulated PTEN expression while suppressing phosphorylation of AKT (p-AKT) and mTOR (p-mTOR). Notably, the PTEN inhibitor reversed both the AKT/mTOR phosphorylation changes and the anti-proliferative/apoptotic effects induced by CKS2 knockdown. Complementary biochemical analyses confirmed the physical interaction between CKS2 and thioredoxin (TXN) through co-immunoprecipitation and immunofluorescence colocalization. TXN knockdown in MM cells resulted in coordinated molecular alterations, including decreased CKS2 expression, elevated PTEN levels, and reduced p-AKT and p-mTOR. Importantly, CKS2 overexpression in TXN-knockdown MM cells rescued these regulatory effects.

CKS2 is a family member of cyclin-dependent kinase subunits (CKS). Richardson *et al.* successfully cloned and identified human CKS2 for the first time in 1990^7^. CKS2, a 9.86 kDa protein comprising 79 amino acid residues, is encoded by a gene localized to chromosome 9q22. This evolutionarily conserved molecule exists as a single splice isoform in humans, with its full-length structure organized into three exons encoding 21 functional domains. Tissue distribution analysis reveals ubiquitous CKS2 expression across multiple physiological systems, including the central nervous, endocrine, respiratory, digestive, urogenital, and musculoskeletal systems, with notable presence in the bone marrow and lymphoid tissues. Immunolocalization studies demonstrate predominant mitochondrial and cytoplasmic localization, complemented by minor nuclear and vesicular distribution.

Emerging evidence positions CKS2 as a critical oncoprotein, with its overexpression strongly correlated with tumor progression and adverse clinical outcomes across various malignancies. Mechanistic studies implicate CKS2 in modulating cell cycle regulation and survival pathways, establishing it as a potential prognostic biomarker and therapeutic target in oncology research. In gastrointestinal malignancies, investigators demonstrate marked upregulation of CKS2 in hepatocellular carcinoma^8^, gastric adenocarcinoma^9^, ^10^, colorectal cancer^11^, and esophageal squamous cell carcinoma^12^ compared to adjacent non-tumor tissues. Similar dysregulation extends to reproductive system neoplasms, with elevated CKS2 levels documented in breast cancer^13^, ^14^, cervical cancer^15^, and prostate cancers^16^, ^17^. Thoracic and neurological malignancies likewise exhibit this pattern, showing aberrant CKS2 expression in non-small cell lung cancer^18^, ^19^ and high-grade gliomas^20^. Notably in breast cancer pathogenesis, Kaplan-Meier survival analysis reveals clinically significant correlations: patients with high CKS2 expression exhibit reduced overall survival, shorter recurrence-free survival, and diminished distant metastasis-free survival compared to low-expression cohorts^13^. Our study revealed significant upregulation of CKS2 in MM patients, with elevated expression correlating strongly with adverse clinical outcomes.

Cancer cells usually have limitless replicative potential and the ability to resist cell death^21^. Apoptosis refers to the orderly and autonomous death of cells controlled by genes to maintain the stability of the internal environment. Unlike cell necrosis, apoptosis is not a passive process, but an active process. Apoptosis is the main barrier of the human body to prevent and suppress cancer, and almost all types of tumor cells can escape apoptosis. In this study, we found that CKS2 can affect the proliferation and apoptosis of MM cells. Knocking down CKS2 inhibits MM cell proliferation and promotes apoptosis while overexpressing CKS2 has the opposite effect. In addition, through the xenograft tumor model, we found that knocking down CKS2 can inhibit MM cell proliferation *in vivo*.

PTEN is a classic tumor suppressor gene located on human chromosome 10q23.3 and is a member of the protein tyrosine phosphatases (PTP) gene family. PTEN participates in the conduction of chemical pathways and can transmit signals to cells, causing them to stop dividing and enter programmed cell death (apoptosis). In addition, PTEN plays an important role in cell growth, adhesion, migration, and infiltration. The researchers found that miR-25-3p can regulate MM cell proliferation and apoptosis through PTEN^22^. Yang, Li-Hui. *et al.* found that lncRNA ANRIL exerts oncogenic function and incubates chemoresistance in MM cells through EZH2-mediated epigenetic silencing of PTEN^23^. Our mechanistic studies demonstrate that CKS2 knockdown in multiple myeloma (MM) cells induces PTEN upregulation with concomitant suppression of AKT/mTOR phosphorylation. PTEN Inhibitor effectively rescued the anti-proliferative effects and apoptotic activation mediated by CKS2 depletion, while restoring AKT/mTOR signaling activity. These findings establish PTEN-mediated regulation of the AKT/mTOR axis as a critical downstream mechanism of CKS2's oncogenic function. Protein interaction analysis revealed that TXN interacts with CKS2. TXN knockdown resulted in the downregulation of CKS2, elevation of PTEN, and downregulation of p-AKT/p-mTOR. Crucially, CKS2 overexpression in TXN-deficient MM cells reversed these molecular alterations, confirming that TXN may be an upstream regulator of CKS2.

In summary, through bioinformatics data analysis, clinical sample verification, and experimental verification, we found that CKS2 is highly expressed in MM patients and is associated with poor prognosis. Depletion of CKS2 significantly inhibited MM cell proliferation and induced apoptosis through PTEN-mediated suppression of AKT/mTOR phosphorylation. TXN interacts with CKS2 and is an upstream regulatory molecule of CKS2. These findings collectively identify CKS2 as a central oncogenic driver in MM pathogenesis and a promising therapeutic target, with its regulatory network involving both upstream TXN modulation and downstream PTEN/AKT/mTOR signaling cascades.

## Supplementary Material

Supplementary figures and tables.

## Figures and Tables

**Table 1:** Correlation analysis between CKS2 and clinical characteristics of patients with multiple myeloma (GSE24080)

Clinical characteristics	CKS2 expression		*P* value
	Low-expression (n=279)	High-expression (n=280)	
Age (year)			
≤60	168	155	0.245
>60	111	125	
Gender			
Female	104	118	0.240
Male	175	162	
Race			
White	249	248	0.799
Other	30	32	
M protein			
IgA	69	64	0.831
IgD	2	1	
IgG	153	160	
Light chain	41	43	
Non-secretory	3	5	
Null	11	7	
ISS stage#			
I	168	126	0.002**
II	14	20	
III	97	133	
β2-M			
mg/l	4.135±4.253	5.341±6.240	0.008**
CRP			
Mg/l	9.183±18.164	14.068±26.774	0.012*
Serum creatinine			
Mg/dl	1.263±1.099	1.383±1.423	0.266
LDH			
U/l	161.108±56.265	182.807±72.830	<0.0001****
Albumin			
g/dl	4.115±0.512	3.983±0.638	0.007**
Hb			
g/dl	11.487±1.766	11.020±1.830	0.002**
MRI ##			
pcs	8.245±12.788	13.743±15.600	<0.0001****
Plasma cell ratio			
(%)	45.162±26.133	47.636±26.410	0.273

#: One patient in the ISS stage was not staged because of missing B2M, so the total number of patients in the ISS staged part was 558. ##: The number of focal lesions (skull, spine, pelvis) defined by magnetic resonance imaging (MRI).

**Table 2:** Cox regression analysis of overall survival in patients with MM (GSE24080)

Clinical characteristics	Univariate COX regression		Multivariate COX regression	
	HR (95% CI)	*P value*	HR (95% CI)	*P value*
Age				
>60 vs ≤60	1.440 (1.067-1.944)	0.017	1.318 (0.971-1.789)	0.077
Gender				
Male vs Female	0.971 (0.716-1.316)	0.848	1.006 (0.739-1.369)	0.972
Race				
Other vs White	0.937 (0.581-1.509)	0.788	1.114 (0.683-1.817)	0.665
ISS Staging				
II vs I	1.222 (0.608-2.453)	0.574	1.179 (0.585-2.376)	0.646
III vs I	2.334 (1.706-3.194)	0.001***	2.163 (1.569-2.981)	0.001***
CKS2 expression				
High vs Low	1.589 (1.170-2.157)	0.003**	1.403(1.028-1.914)	0.033*

## References

[1] Cowan AJ, Green DJ, Kwok M, Lee S, Coffey DG, Holmberg LA (2022). Diagnosis and Management of Multiple Myeloma: A Review. JAMA.

[2] Kyle RA, Rajkumar SV (2008). Multiple myeloma. Blood.

[3] Callander NS, Baljevic M, Adekola K, Anderson LD, Campagnaro E, Castillo JJ (2022). NCCN Guidelines(R) Insights: Multiple Myeloma, Version 3.2022. J Natl Compr Canc Netw.

[4] Soekojo CY, Chng WJ (2022). Treatment horizon in multiple myeloma. Eur J Haematol.

[5] Rajkumar SV, Dimopoulos MA, Palumbo A, Blade J, Merlini G, Mateos MV (2014). International Myeloma Working Group updated criteria for the diagnosis of multiple myeloma. Lancet Oncol.

[6] Jing Z, Yu W, Li A, Chen X, Chen Y, Chen J (2022). Trifluoperazine Synergistically Potentiates Bortezomib-Induced Anti-Cancer Effect in Multiple Myeloma via Inhibiting P38 MAPK/NUPR1. Tohoku J Exp Med.

[7] Richardson HE, Stueland CS, Thomas J, Russell P, Reed SI (1990). Human cDNAs encoding homologs of the small p34Cdc28/Cdc2-associated protein of Saccharomyces cerevisiae and Schizosaccharomyces pombe. Genes Dev.

[8] Zhang J, Song Q, Liu J, Lu L, Xu Y, Zheng W (2019). Cyclin-Dependent Kinase Regulatory Subunit 2 Indicated Poor Prognosis and Facilitated Aggressive Phenotype of Hepatocellular Carcinoma. Dis Markers.

[9] Zhou Y, Zeng J, Zhou W, Wu K, Tian Z, Shen W (2022). Prognostic significance of CKS2 and CD47 expression in patients with gastric cancer who underwent radical gastrectomy. Scand J Immunol.

[10] Tanaka F, Matsuzaki S, Mimori K, Kita Y, Inoue H, Mori M (2011). Clinicopathological and biological significance of CDC28 protein kinase regulatory subunit 2 overexpression in human gastric cancer. Int J Oncol.

[11] Yu MH, Luo Y, Qin SL, Wang ZS, Mu YF, Zhong M (2015). Up-regulated CKS2 promotes tumor progression and predicts a poor prognosis in human colorectal cancer. Am J Cancer Res.

[12] Kita Y, Nishizono Y, Okumura H, Uchikado Y, Sasaki K, Matsumoto M (2014). Clinical and biological impact of cyclin-dependent kinase subunit 2 in esophageal squamous cell carcinoma. Oncol Rep.

[13] Huang N, Wu Z, Hong H, Wang X, Yang F, Li H (2019). Overexpression of CKS2 is associated with a poor prognosis and promotes cell proliferation and invasion in breast cancer. Mol Med Rep.

[14] Wang J, Xu L, Liu Y, Chen J, Jiang H, Yang S (2014). Expression of cyclin kinase subunit 2 in human breast cancer and its prognostic significance. Int J Clin Exp Pathol.

[15] Qin L, Luo X, Qin X, Huang H, Zhang L, Chen S (2022). Comprehensive Expression Profiling and Molecular Basis of CDC28 Protein Kinase Regulatory Subunit 2 in Cervical Cancer. Int J Genomics.

[16] Liang X, Huang R, Ping X, Deng W, Xiang S, Wang Z (2024). Upregulation of CKS2 in immunosuppressive cells is associated with metastasis and poor prognosis in prostate cancer: a single-cell RNA-sequencing analysis. Transl Cancer Res.

[17] Lan Y, Zhang Y, Wang J, Lin C, Ittmann MM, Wang F (2008). Aberrant expression of Cks1 and Cks2 contributes to prostate tumorigenesis by promoting proliferation and inhibiting programmed cell death. Int J Cancer.

[18] Feng J, Hu M, Li Z, Hu G, Han Y, Zhang Y (2022). Cyclin-Dependent Kinase Subunit 2 (CKS2) as a Prognostic Marker for Stages I-III Invasive Non-Mucinous Lung Adenocarcinoma and Its Role in Affecting Drug Sensitivity. Cells.

[19] Wan Z, Wang L, Yang D, Li P, Liu Q, Wang B (2022). CKS2 Promotes the Growth in Non-Small-Cell Lung Cancer by Downregulating Cyclin-Dependent Kinase Inhibitor. Pathobiology: journal of immunopathology, molecular and cellular biology.

[20] Feng F, Zhao Z, Cai X, Heng X, Ma X (2022). Cyclin-dependent kinase subunit2 (CKS2) promotes malignant phenotypes and epithelial-mesenchymal transition-like process in glioma by activating TGFbeta/SMAD signaling. Cancer Med.

[21] Hanahan D, Weinberg RA (2011). Hallmarks of cancer: the next generation. Cell.

[22] Zi Y, Zhang Y, Wu Y, Zhang L, Yang R, Huang Y (2021). Downregulation of microRNA-25-3p inhibits the proliferation and promotes the apoptosis of multiple myeloma cells via targeting the PTEN/PI3K/AKT signaling pathway. Int J Mol Med.

[23] Yang LH, Du P, Liu W, An LK, Li J, Zhu WY (2021). LncRNA ANRIL promotes multiple myeloma progression and bortezomib resistance by EZH2-mediated epigenetically silencing of PTEN. Neoplasma.

