# Supplementary material for: CKS2 Silencing Affects Proliferation and Apoptosis in Multiple Myeloma through the PTEN/ AKT/mTOR Pathway
Source: J Cancer. 2025 Mar 3;16(6):1987–2000. doi: 10.7150/jca.106190 (PMC11905417; doi:10.7150/jca.106190)
Supplement: Supplementary file 1 — Supplementary figures and tables. [file jcav16p1987s1.pdf]

Supplement Table 1. Expression of CKS2 in clinical characteristic subgroups of multiple myeloma patients (clinical specimens)

Clinical parameters	number	CKS2 expression	*P* value
Age（year）			
≤60	17	2.051±1.287	0.170
＞60	27	1.617±0.780	
Gender			
Female	24	1.964±1.108	0.204
Male	20	1.571±0.870	
M protein			
IgA	4	2.123±2.229	0.337
IgG	24	1.924±0.941	
Light chain	16	1.492±0.668	
ISS stage			
I	11	1.157±0.432	0.040*
II	15	1.837±0.747	
III	18	2.125±1.286	

Supplement Table 2. Correlation analysis between CKS2 and clinical characteristics of patients with multiple myeloma.

Clinical characteristics	CKS2 expression		*P value*
	Low-expression（n=22）	High-expression（n=22）	
Age（year）			
≤60	7	10	0.353
＞60	15	12	
Gender			
Female	10	14	0.226
Male	12	8	
M protein			
IgA	3	1	0.168
IgG	9	15	
Light chain	10	6	
ISS stage			
I	9	2	0.042*
II	5	10	
III	8	10	
β2M			
(mg/L)	7.507±9.063	8.220±7.122	0.773
Serum creatinine			

(umol/L)	176.727±198.313	218.727±233.566	0.524
LDH			
(U/L)	189.955±72.287	259.773±208.366	0.134
Albumin			
(g/L)	37.818±7.035	35.136±6.693	0.202
Hb			
(g/L)	98.364±36.372	87.000±25.124	0.235
Plasma cell ratio			
(%)	21.330±7.523	26.880±12.416	0.080

Supplement Table 3. mRNA expression of 14 factors that may interact with CKS2 in GSE6477 and GSE47552

GSE6477						
GENE	logFC	AveExpr	t	P.Value	adj.P.Val	B
TXN	1.266464	11.42614	6.330219	9.38E-09	5.32E-07	9.736964
RFC5	0.012152	7.749798	0.062149	0.950582	0.971479	-6.74245
BUB3	0.323208	11.29508	2.639837	0.009777	0.037544	-3.39891
RACGAP1	0.325298	7.653636	1.488736	0.140054	0.272394	-5.6527
RAN	0.963144	11.25261	6.248251	1.35E-08	7.19E-07	9.381941
CCNA2	-0.50732	7.864876	-1.89553	0.061232	0.15076	-4.9879
UBE2C	0.160636	9.632893	1.349812	0.180463	0.327396	-5.84497
CKS1B	0.324901	9.394299	1.713541	0.090057	0.199259	-5.30382
PTTG1	0.516713	10.77779	2.908942	0.004568	0.021207	-2.71386
CDC20	-0.31274	7.521406	-1.0273	0.307035	0.47376	-6.2212
KPNA2	0.316456	9.994538	2.187555	0.031294	0.091664	-4.42009
CDC7	0.448237	7.813533	1.969246	0.052002	0.133487	-4.85157
RPA3	2.000149	9.601966	8.056523	3.12E-12	5.72E-10	17.51766
ILF2	0.191178	10.56314	1.446559	0.151495	0.288243	-5.71297

GSE47552						
GENE	logFC	AveExpr	t	P.Value	adj.P.Val	B
TXN	1.237379	9.676092	4.545328	3.82E-05	0.000969	1.994222
RFC5	-0.41809	5.116462	-2.86859	0.00615	0.039491	-2.75178
BUB3	-0.12421	8.01278	-0.58741	0.559728	0.704113	-6.36656
RACGAP1	-0.28607	5.665759	-1.16158	0.251252	0.427936	-5.87335
RAN	0.166834	9.274707	0.905061	0.370035	0.545479	-6.13241
CCNA2	-0.51502	6.064635	-1.89566	0.064144	0.178542	-4.80779
UBE2C	0.312557	5.882292	2.430805	0.018922	0.082353	-3.75785
CKS1B	0.324083	7.495113	2.255361	0.028792	0.107838	-4.12523
PTTG1	0.523916	6.871898	1.619184	0.112079	0.255851	-5.26315
CDC20	-0.32434	5.470872	-2.37255	0.021797	0.090629	-3.88224
KPNA2	1.108177	8.905538	4.280871	9.06E-05	0.001822	1.175184
CDC7	-0.0039	3.648184	-0.01998	0.984141	0.99129	-6.5383
RPA3	0.254629	6.216429	2.096018	0.041475	0.136	-4.43968
ILF2	-0.04631	9.698966	-0.25257	0.801695	0.874905	-6.50661
